# Efficiency Analysis of Healthcare System in Lebanon Using Modified Data Envelopment Analysis

**DOI:** 10.1155/2018/2060138

**Published:** 2018-07-02

**Authors:** Mustapha D. Ibrahim, Sahand Daneshvar

**Affiliations:** Department of Industrial Engineering, Eastern Mediterranean University, North Cyprus, via Mersin 10, Turkey

## Abstract

The inflow of refugees from Syria into Lebanon necessitates a robust and efficient healthcare system in Lebanon to withstand the growing demand for healthcare service. For this purpose, we evaluate the efficiency of healthcare system in Lebanon from 2000 through 2015 by applying a modified data envelopment analysis (DEA) model. We have selected four output variables: life expectancy at birth, maternal mortality ratio, infant mortality rate, and newly infected with HIV and two input variables: total health expenditure (% of GDP) and number of hospital beds. The findings of the paper show improvement in the efficiency of the healthcare system in Lebanon after the widespread of the health system reform in 2005. It also shows that reduction in health expenditure does not necessarily reduce efficiency if operational and technical aspect of the healthcare system is improved. The study infers that the healthcare system in Lebanon is capable of withstanding the increase in health demand provided further resources are made available and the existing technical and operational improvement are maintained.

## 1. Introduction

The improvement of a population's health is dependent on an equitable and efficient healthcare system [1]. Productivity of citizens depends on their health level, and higher rate of economic growth can be influenced by a more efficient healthcare system [2]. In economies with both high development index and medium development index, increase in efficiency of expenditure seems to be the only way public healthcare systems can overcome the pressure of expenditure [3]. Identifying areas of improvement in the healthcare system necessitates evaluating the efficiency of the existing system. Evaluation of healthcare system is a complex process due to limited data and methodological complications. The health status of the citizens has to be identified, which is influenced by their socioeconomic stability, productivity level, and welfare level [1]. Researches are becoming increasingly focused on the efficiency of the healthcare system. Hellingsworth, in his review of 317 references on the efficiency of healthcare delivery in the context of frontier analysis, shows that only a few performed the analysis on the basis of macroperspective, which gives an insight into the overall healthcare system performance [4].

The healthcare service of societies has improved impressively over the past couple years, more so for the services provided for maternal and infant health, and improving live expectancy. However, the increase of healthcare outcomes at national level may not necessarily translate to improvement of the indicators for some of the citizens who are predisposed to sicknesses. In addition, the progress of some cases is stagnant, and the improvements are insignificant. In this context, the efficiency of financial infrastructure and human resources in the health sector across Lebanon is a relevant topic for researchers and health policy makers.

The growing concern for the efficiency of the healthcare system in Lebanon is motivated by the inflow of refugees from Syria. According to the United Nations High Commissioner for Refugees (UNHCR), there are more than 1 million Syrian refugees in Lebanon, 23% women and 51.6% are children, 18% of which are under five years old. The needs of the refugees have had enormous impact on the public finance of Lebanon, increasing Government expenditure in public services and compounding the negative economic consequences of the regional instability. The current humanitarian system has contributed in increasing the fragmentation of the Lebanese health system [5], recognizing the fact that healthcare is needed and available resources to satisfy the growing demand is limited. Going forward, it is important to maintain a viable healthcare system to satisfy the citizens and refugees. Efficiency improvement, cost reduction, and introduction of new technology will contribute to that effect [6]. Therefore, it is imperative for the healthcare system of Lebanon to operate efficiently in order to handle the increase in healthcare demand. The health sector priorities as identified by the national health policy include health service delivery and strengthening the role of the ministry of public health as the principal steward. Providing universal health coverage to the national residents is a considerable step towards reform at the social level [7].

The healthcare system in Lebanon is diversified with public and private healthcare providers, financiers, political agenda, and various laws and regulations [8]. National Social Security Fund (NSSF) was established in 1963 to provide employees and their dependents with national insurance coverage for work-related incidents, maternity, sickness, and diseases. The Ministry of Health financing scheme insures the uninsured; the beneficiaries are majority of the population, about (42.7%). The scheme is funded by the government budget and covers 80% of the hospital bills as the direct payment with absolute coverage of expensive intervention. Prior to 1975, Lebanon was the center for the entire region's healthcare because of its advance healthcare services and medical institutions. The 1975 civil war caused enormous problems, and the treatment of traumatic injuries overwhelmed the health sector. The health sector declined rapidly and was taken over by the private sector and nongovernmental organizations (NGOs). Similar case can be made with the growing number of refugees from Syria, hence the urgent need for a performance evaluation of the existing system and proper improvement strategies for sustaining efficient services. The overall objective of this paper is to evaluate efficiency and present efficiency improvement options of the healthcare system in the context of finance, infrastructure, and medical indicators. Lebanon has one of the most expensive healthcare systems in the world [9]. Lebanon has a high out of pocket health expenditure which leads to exposure of households to financial risk as a result of ill health [10]. And the minimum public expenditure to primary healthcare compared to secondary and tertiary health brings more burden to Lebanese and vulnerable population in particular. A performance measure of the health system in eastern Mediterranean region ranks Lebanon as 17 out of 21 countries in health production and determinants [11]. Ammar [12] presented a document (health reform in Lebanon) stating the 12 key achievements of the ministry of public health over a 10-year period. The document points out an increase in performance of the healthcare system, with improvement in supply of human resources, strength of the primary healthcare system, quality and accreditation improvement, and autonomy of public hospitals among others. The aim of this study is to create some idea as to the present state of the healthcare system in the country in the context of the available selected variables. Most of the studies that are reviewed used life expectancy at birth as output and health expenditure as inputs [11]. Other studies such as Alfonso and St. Aubyn [13] used number of beds and health employment as inputs. In a more recent study, Asandului et al. [1] and Medeiros and Schwierz [14] analyzed the entire European states' healthcare system, the list of variables used were the number of hospital beds per 1000, number of physicians per 1000, health expenditure % of GDP, life expectancy, adjusted life expectancy, infant mortality, and health expenditure per capita. In this paper, similar indicators are used to estimate the performance of the healthcare system in Lebanon.

To ensure universal health coverage for the vulnerable Lebanese, Syrians, and Palestine refugees, the Lebanese healthcare system needs to perform efficiently [5]. Getting the data on the health status of the refugees is a challenging task. However, the performance of the Lebanese healthcare system should show their ability to withstand the increasing pressure as a result of the refugees' impact on the existing system. Consequently, improving the efficiency and operations of the existing healthcare system would impact the healthcare services delivered to the refugees.

## 2. Method

### 2.1. Data Envelopment Analysis

This paper utilizes the data envelopment analysis (DEA) method developed by Charnes et al. [15] to evaluate the efficiency of the healthcare system in Lebanon. It is a nonparametric linear programming method that estimates the efficiency frontier of evaluated units known as decision-making units (DMUs). Emrouznejad and Dey [16] highlighted the use of frontier methodologies and multicriteria decision-making for performance measurement in the health sector. Cheng and Zervopoulos [17] evaluated health system using directional distance function in DEA. Their analysis incorporates both desirable and undesirable outputs of a health system.

In this paper, in order to evaluate the relative efficiency of the healthcare system in Lebanon over a 16-year period (2000–2015), a modified DEA model developed by Daneshvar et al. [18] is employed. The modified DEA model pays special attention to the weak efficient and highly inefficient units. It proposes efficiency scores that do not exaggerate the performance of the units at the weak part of the frontier under variable return to scale (VRS) assumption. The modified DEA model used is as follows.

Consider *n*DMUs (years in our case) with each DMU *j*(*j*=1,…, *n*) using *m* inputs *x*_*j*_=(*x*_1*j*_, *x*_2*j*_,…, *x*_*mj*_) > 0 to produce *s* outputs*y*_*j*_=(*y*_1*j*_, *y*_2*j*_,…, *y*_*sj*_) > 0. The best weights for the variables are *u*_*r*_ for outputs and *v*_*i*_ for inputs. *y*_*r*0_ and *x*_*i*0_ are the input and output for a particular DMU *j* under evaluation. The steps for applying the modified DEA model is as follows: first use model (1) on the entire DMUs, and then use model (2) on only the DMUs with score = 1 identified by model (1). Use (3) to find the upper bound for *u*_0_, in our case *φ*^*∗*^=0.77. Finally, apply model (4) on the entire DMUs to get the efficiency scores for each DMU:

(1) $$\begin{array}{c} \max\;\overset{s}{\underset{r=1}{\sum }}u_{r}y_{r0}+u_{0}\\ \text{subject to}\;\overset{m}{\underset{i=1}{\sum }}v_{i}x_{i0}=1\\ \overset{s}{\underset{r=1}{\sum }}u_{r}y_{rj}-\overset{m}{\underset{i=1}{\sum }}v_{i}x_{ij}+u_{0}\leq 0,\;j=1\ldots n,\\ u_{r}\geq 0,\\ v_{i}\geq 0,\\ u_{0}\text{ free}. \end{array}$$

(2) $$\begin{array}{c} \varphi =\max\;u_{0}\\ \text{subject to}\;\overset{s}{\underset{r=1}{\sum }}u_{r}y_{r0}+u_{0}=1\\ \overset{m}{\underset{i=1}{\sum }}v_{i}x_{i0}=1,\\ \overset{s}{\underset{r=1}{\sum }}u_{r}y_{rj}-\overset{m}{\underset{i=1}{\sum }}v_{i}x_{ij}+u_{0}\leq 0,\\ u_{r}\geq 0,\\ v_{i}\geq 0,\\ u_{0}\text{ free}.\\ \text{subject to}\;\overset{m}{\underset{i=1}{\sum }}v_{i}x_{i0}=1\\ \overset{s}{\underset{r=1}{\sum }}u_{r}y_{rj}-\sum_{i=1}^{m}v_{i}x_{ij}+u_{0}\leq 0,\;j=1\ldots n,\\ u_{r}\geq 0,\\ v_{i}\geq 0,\\ u_{0}\leq \varphi^{*}. \end{array}$$

(3) $$\begin{array}{c} \varphi^{*}=\max\left\{\left.\varphi \right|\;\varphi \neq 1\text{ for efficient units}\right\}.\\ \text{subject to}\;\overset{m}{\underset{i=1}{\sum }}v_{i}x_{i0}=1\\ \overset{s}{\underset{r=1}{\sum }}u_{r}y_{rj}-\sum_{i=1}^{m}v_{i}x_{ij}+u_{0}\leq 0,\;j=1\ldots n,\\ u_{r}\geq 0,\\ v_{i}\geq 0,\\ u_{0}\leq \varphi^{*}. \end{array}$$

(4) $$\begin{array}{c} \max\;\overset{s}{\underset{r=1}{\sum }}u_{r}y_{r0}+u_{0}\\ \text{subject to}\;\overset{m}{\underset{i=1}{\sum }}v_{i}x_{i0}=1\\ \overset{s}{\underset{r=1}{\sum }}u_{r}y_{rj}-\sum_{i=1}^{m}v_{i}x_{ij}+u_{0}\leq 0,\;j=1\ldots n,\\ u_{r}\geq 0,\\ v_{i}\geq 0,\\ u_{0}\leq \varphi^{*}. \end{array}$$

### 2.2. Efficiency Analysis

Each year (2000 to 2015) is referred to as the decision-making units (DMUs) and included in the analysis as shown in Table 1. We utilized an input-orientation model. The input-orientation model is appropriate in this context because the inputs are assumed to be at the discretion of the health industry and should be properly utilized to achieve the best possible outcome. This analysis provides policy makers and general public with useful information regarding the state of the healthcare system and provides improvement in focus of health outcomes for proper utilization of resources. Four outputs were chosen for the efficiency model: life expectancy at birth, maternal mortality ratio, infant mortality rate, and people newly infected with HIV. Life expectancy at birth is a robust healthcare system outcome used widely in the literature. The effect of increase in health expenditure that has on life expectancy was accentuated by [19]. It is considered to be one of the primary indicators of the healthcare system efficiency of a country. It is confirmed by international studies as an output variable used to asses efficiency of healthcare systems [20]. As for the maternal mortality ratio, infant mortality rate, and people newly infected with HIV, which are also seldom used in the literature as outputs are negative outputs, since the DEA technique is applied in such a way that “more is better,” the inverse of the data is used in the efficiency analysis, thus satisfying the more is better approach by converting the largest number to the smallest, and vice versa [1, 14, 21–23]. The inputs included in the analysis are as follows: health expenditure total (% of GDP) and hospital beds (per 1,000 people), which are commonly used in this context and accepted by the conceptual model that recognizes the following determinants on individuals health and available medical services and environment.  Table 2 presents the definition of inputs and outputs used in the DEA model. The data used for the analysis are extracted from the World Bank database under the world development indicator (http://databank.worldbank.org/data/reports.aspx?source=world-development-indicators). Using the modified DEA model (1 to 4), with the WinQSB linear programming software 2.0, the efficiency of the healthcare system is analyzed.

## 3. Results

**Figure 1:**
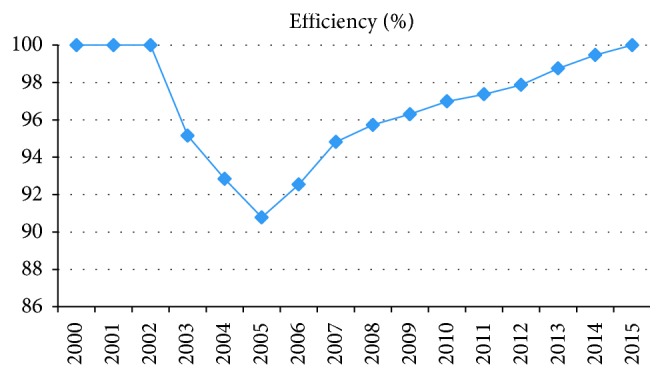
Efficiency description.

Table 3 shows the efficiency score for each year from 2000 to 2015; 100% signifies the year as efficient and anything less as inefficient. The result of the DEA model reveals that the overall efficiency of the healthcare system in Lebanon is inefficient, with only 4 years (2000, 2001, 2002, and 2015) as efficient over the 16-year period, with an efficiency average of 96.79%. As can be seen from Figure 1, there is a sharp decrease in efficiency from 2003 to 2005. This can be attributed to the increase in the number of newly infected HIV and maternal mortality ratio. The increase in the number of hospital beds per 1000 does not reflect significantly on the infant mortality rate despite its improvement. However, the efficiency improved constantly from 2006 to 2015. The improvement in efficiency can be attributed to the enhanced health sector reform project, in addition to strengthening other aspects of administrative and technical nature of the health sector. Furthermore, the introduction of information technology via automation data collection and shearing to ensure transparency in public financing contributes to efficiency improvement [24]. The development of the visa system and its coverage of the entire Lebanon in February 2005 linking all data bases are among the major achievements in the healthcare system [12]. The data show a continuous decrease in health expenditure, total (% of GDP), from 2008 to 2015; however, the efficiency continues to show steady improvement. This finding contradicts the hypothesis of [25], which states that an increase in public health expenditure would automatically lead to an improvement in self-estimated health status. Our analysis shows that improvement in the healthcare system has more to do with policy reforms and proper implementation than increasing health expenditure. Investing in preventive treatments and proper technology can be an important factor in improving healthcare system efficiency. It can therefore be concluded that the healthcare system in Lebanon utilizes their financial resources efficiently, and further investment in this indicator can have significant impact on the health outcomes if proper utilization of the financial resources is maintained.

Weight of a variable in DEA signifies the level of contribution of that variable to the efficiency of a DMU. The weight distribution across the entire DMUs gives a general idea as to which variable contributes the most to efficiency. The weight distribution of the variables used in the efficiency evaluation shows that (*x*2) hospital beds (per 1,000 people) and (*y*1) life expectancy at birth total (years) contribute the most to efficiency, given their high average values and distribution. Therefore, improving the utilization of the number of beds and increasing the life expectancy at birth will affect the efficiency significantly. (*x*1) Health expenditure, total (% of GDP), also has significant impact on the efficiency of the healthcare system.

## 4. Discussion

In this paper, we developed a modified data envelopment analysis (DEA) model for assessing the efficiency of the healthcare system in Lebanon and to infer if the healthcare system is capable of handling the increase in demand of healthcare services due to the growing number of refugees. The main findings of this paper suggest that the healthcare system in Lebanon is improving its efficiency and continuous to do so. A study of antenatal care among Syrian refugees in Lebanon [26] concluded that the standards of antenatal care are not being met for pregnant Syrian refugee women in Lebanon. Their study suggests increase in antenatal care visits and improvement in early testing and interventions to improve pregnancy outcomes. However, the number of maternal deaths in Lebanon continues to decrease significantly over the years by about 13% in 2015 (Information on number of maternal deaths in Lebanon, world development indicator. World Bank http://databank.worldbank.org/data/reports.aspx?source=world-development-indicators). In addition, the out of pocket health expenditure (% of total expenditure on health) has decreased from 42.85% in 2005 to 36.42% in 2014. These show the competency of the healthcare system to improve services provided for the refugees if more resources are made available for the healthcare system. In terms of funding and the ability of the Lebanese healthcare system to withstand the increase in demand of services as a result of the refugees, further investment in the healthcare system would yield a positive outcome, as the analysis has shown that the healthcare system continues to improve even with the decrease in health expenditure. Our study shows improvement in utilization of available resources by the healthcare system. The improvement will continue to be maintained if the technical and operational service of the healthcare system during the healthcare system reform is maintained. Our analysis was based on a time series data over a 16-year period and hence does not pretend to provide a definitive conclusion regarding the overall efficiency level of the country. However, it presents an overview of the system's performance based on the selected and available indicators.

## Figures and Tables

**Table 1:** Efficiency data.

Years	DMUs	*x*1	*x*2	*y*1	*y*2	*y*3	*y*4
2000	1	10.8616	2.93	74.4317	42	17.1	100
2001	2	10.9044	3	74.9221	39	16.1	100
2002	3	10.0465	3.15	75.4147	36	15.1	100
2003	4	9.30239	3.3	75.9033	33	14.1	200
2004	5	8.90981	3.45	76.3801	30	13.1	200
2005	6	8.41981	3.6	76.8326	27	12.1	200
2006	7	8.83001	3.515	77.2467	24	11.2	200
2007	8	8.90376	3.43	77.6159	23	10.4	200
2008	9	8.07053	3.465	77.9392	21	9.7	200
2009	10	7.42449	3.5	78.2195	20	9.2	200
2010	11	7.19134	3.5	78.4654	19	8.7	200
2011	12	7.12493	3.5	78.6899	18	8.3	200
2012	13	6.99093	3.5	78.9085	17	7.9	200
2013	14	6.63325	3.5	79.1337	16	7.6	200
2014	15	6.39371	3.5	79.3731	16	7.3	200
2015	16	6.27	3.5	79.6286	15	7.1	200

*x*1: health expenditure, total (% of GDP); *x*2: hospital beds (per 1,000 people); *y*1: life expectancy at birth, total (years); *y*2: maternal mortality ratio (modeled estimate, per 100,000 live births); *y*3: mortality rate, infant (per 1,000 live births); *y*4: adults (aged 15+) and children (aged 0–14) newly infected with HIV.

**Table 2:** Definition of variable used in the DEA model.

Variable	Role	Definition
Health expenditure, total (% of GDP)	Input (*x*1)	The sum of public and private health expenditure which covers health service provision (preventive and curative) but does not include provision of water and sanitation.
Hospital beds (per 1,000 people)	Input (*x*2)	Hospital beds include inpatient beds available in public, private, general, and specialized hospitals and rehabilitation centers.
Life expectancy at birth, total (years)	Output (*y*1)	Life expectancy at birth indicates the number of years a newborn infant would live if prevailing patterns of mortality at the time of its birth were to stay the same throughout its life.
Maternal mortality ratio		—
Modeled estimate (per 100,000 live births)	Output (*y*2)	Maternal mortality ratio is the number of women who die from pregnancy-related causes while pregnant or within 42 days of pregnancy termination.
Mortality rate, infant (per 1,000 live births)	Output (*y*3)	Infant mortality rate is the number of infants dying before reaching one year of age.
Adults (aged 15+) and children (aged 0–14) newly infected with HIV	Output (*y*4)	Number of adults (aged 15+) and children (aged 0–14) newly infected with HIV.

**Table 3:** Efficiency scores.

Years	Efficiency (%)
2000	100
2001	100
2002	100
2003	95.15
2004	92.84
2005	90.78
2006	92.54
2007	94.82
2008	95.73
2009	96.3
2010	96.99
2011	97.37
2012	97.87
2013	98.75
2014	99.47
2015	100
Mean.	96.79
